# The Gums; with Late Discoveries on Their Structure, Growth, Connections, Diseases, and Sympathies

**Published:** 1841-12

**Authors:** 


*The Gums; with late discoveries on their structure, growth, connections, diseases, and sympathies.* By George Waite, Member of the London Royal College of Surgeons. London, 1835.

It is not perhaps generally known by the dental profession, that the work, bearing the above title, has been republished in this country. It constitutes one of the series of Dr. Bell's "*Select Medical Library,*" and was published by Messrs. Haswell, Barrington and Haswell, Philadelphia, in 1838. We mention this, because the work is one of value, and that our professional brethren may know where it can be had.

The structure, diseases, &c. of the gums, are treated on by Mr. Waite in a very able manner, and more at length, than by any preceding writer on the subject. It is a work which every surgeon-dentist should have, and had we the time we should be glad to give a more extended notice of it, but as we have not, we must be content with thus merely inviting the attention of the profession to it.—*Balt. Ed.*

### Recent Publications.

